# Multivariate Nature of Fish Freshness Evaluation by Consumers

**DOI:** 10.3390/foods11142144

**Published:** 2022-07-20

**Authors:** Fernanda M. Viana, Maria Lucia G. Monteiro, Rafaela G. Ferrari, Yhan S. Mutz, Inayara B. A. Martins, Ana Paula A. A. Salim, Marcela De Alcantara, Rosires Deliza, Sérgio B. Mano, Carlos A. Conte-Junior

**Affiliations:** 1 Instituto de Química, Universidade Federal do Rio de Janeiro, Av. Athos da Silveira Ramos, 149, Rio de Janeiro 21949-909, RJ, Brazil; fernanda_medeiros@msn.com (F.M.V.); rafaelaferrari@yahoo.com.br (R.G.F.); yhan.mutz@hotmail.com (Y.S.M.); carlosconte@hotmail.com (C.A.C.-J.); 2 Departamento de Tecnologia de Alimentos, Faculdade de Veterinária, Universidade Federal Fluminense, Rua Vital Brazil Filho, 64, Niterói 24230-340, RJ, Brazil; apaula15br@yahoo.com.br (A.P.A.A.S.); sergiomano@id.uff.br (S.B.M.); 3 Núcleo de Análise de Alimentos (NAL), LADETEC, Universidade Federal do Rio de Janeiro, Av. Horácio Macedo, 1281, Rio de Janeiro 21941-598, RJ, Brazil; 4 Departamento de Tecnologia de Alimentos, Universidade Federal Rural do Rio de Janeiro, BR 456, km 7, Seropédica 23897-000, RJ, Brazil; inayarabeatriz@yahoo.com.br; 5 PDJ-CNPq/Embrapa Agroindústria de Alimentos, Av. das Américas, 29501, Rio de Janeiro 23020-470, RJ, Brazil; marceladealcantara@gmail.com; 6 Embrapa Agroindústria de Alimentos, Av. das Américas, 29501, Rio de Janeiro 23020-470, RJ, Brazil; rosires.deliza@embrapa.br; 7 Instituto Nacional de Controle de Qualidade em Saúde, Fundação Oswaldo Cruz, Av. Brasil, 4365, Rio de Janeiro 21040-900, RJ, Brazil

**Keywords:** survival analysis, sensory shelf life, quality indicators, socioeconomic profiles, chemometric analysis

## Abstract

The aim of the present study was to evaluate the sensory acceptability limit of refrigerated fish through a multivariate approach, involving classic physicochemical and bacteriological indicators and considering different consumer profiles. The results of the survival analysis demonstrated that, in general, consumers still considered the fish to be suitable for purchase (4.128 days of storage), despite being microbiologically unsuitable for consumption. However, the subsequent division of consumers into clusters indicated that women and individuals with high income and education levels tend to reject fish with few days of storage (3.650 days), mainly due to discoloration, despite still being microbiologically suitable for consumption. Thus, these segments present a safer behavior regarding the purchase of fresh fish. The influence of different frequencies of fish consumption and age of consumers on the assessment of fish freshness was not clarified. The responsibility for ensuring safe and healthy products at the point of sale must lie with the producers and distributors. However, improving consumers’ ability to make good choices when buying fresh fish would bring social and economic benefits related to public health and to the seafood industry, because it would enable them to make relevant claims and demand their rights.

## 1. Introduction

Fish is rich in nutrients related to health benefits; however, its high perishability negatively impacts the global average consumption, in addition to representing the main reasons of economic loss and waste generation towards the fish production chain [1,2]. Consumers usually evaluate fish quality and freshness based on its appearance at the point of sale, because fresh fish is frequently sold in clear packaging [3,4,5]. Nonetheless, changes in sensory attributes such as color and texture occur progressively due to multiple microbial and physicochemical processes, which lead consumers to have difficulties and uncertainties when conducting freshness assessments [6]. Freshness misjudgment not only affects the acceptance of food products, but can also lead to malnutrition, health problems and/or food waste [7,8].

The determination of freshness and acceptability limits are crucial to industry and consumers, because they influence the commercial value of fresh fish. Previous studies [9,10] observed that some classes of consumers are willing to pay more for high-quality attributes. Furthermore, the decision to consume or reject food depends not only on its intrinsic quality attributes, but on a combination of intrinsic and extrinsic factors [11,12]. For instance, Østli et al. [13] reported that elder consumers tended to choose fresher fish to buy when compared with younger consumers. Therefore, affective, cognitive, and behavioral components also influence the final purchase decision, which may differ between socioeconomic groups [14,15] and can influence the development of marketing strategies.

To assess consumers’ acceptability limits for fish over storage, many consumer tests have adopted pre-established scoring scales. However, they do not always reflect the consumer’s decision to accept or reject the product. Thus, acceptability limits can be better assessed by the survival analysis methodology, where a risk function capable of describing product rejection during storage is obtained through consumer responses [16]. Such methodology has been extensively applied to estimate the sensory shelf life of several food products such as dairy [17], bakery [18,19] and vegetables [20,21]. To the best of our knowledge, survival analysis has only been applied once in fish; however, it was used as a single freshness indicator for determining the shelf life of Atlantic cod (*Gadus morhua*) stored in ice [13].

Similarly, assessments of fish quality and safety by unique physicochemical and/or microbiological indicators are hampered by the multifactorial characteristics of the degradative processes. Despite the advances in developing novel techniques for evaluating fish freshness, there are still many challenges on this matter. In fact, some methodologies may be limited by their high cost and delay in obtaining satisfactory results [2,22]. Moreover, it is difficult to choose a limiting indicator because its relevance may also vary according to fish species, storage procedures and processing conditions [23,24]. Considering these facts, it is important to assess the relationship between the most common analytical freshness parameters and the acceptability limits defined by consumers. From an appropriated assessment, it is possible to observe which parameters influence the consumers’ rejection decision and whether this decision is, in general, reliable and safe.

In this context, the present research aimed to: (i) determine the consumers’ acceptability limit for the purchase and consumption of refrigerated Nile tilapia (*Oreochromis niloticus*) fillets through survival analysis; (ii) identify the relationship between consumers’ purchase rejection with analytical freshness parameters; and (iii) investigate the influence of consumers’ socioeconomic characteristics on the acceptability limit for purchasing the refrigerated Nile tilapia (*Oreochromis niloticus*) fillets.

## 2. Materials and Methods

### 2.1. Material and Experimental Design

This study was approved by the Ethics Committee of the University Hospital Clementino Fraga Filho at UFRJ, Brazil (No. 27822620.9.0000.5257).

The Nile tilapia (*Oreochromis niloticus*) was chosen for our study because it is one of the most cultivated species globally and presents great popularity among consumers [1]. Moreover, refrigerated storage is possibly the simplest method of preserving fish for a short time and, despite its low efficiency, it is still preferred by consumers. Indeed, consumers seem to relate high levels of processing with a loss of quality, safety, and healthiness [25,26].

The present study used a reverse storage design in order to allow the consumers to evaluate all samples in a single session at the end of the total storage period [27]. Therefore, the fish samples were collected from the same producer at the same conditions, at each previously established time point of analysis (days 8, 6, 4, 2 and 0). A total mass of 15 kg of fresh Nile tilapia (*Oreochromis niloticus*) fillets (192.60 ± 4.39 g each) was purchased from a local fish farm in Rio de Janeiro, Brazil. On each day of purchase, 16 fillets were immediately transported on filtered ice (0 °C) in Styrofoam boxes to the laboratory. Then, 10 fillets were randomly divided to form five different repeats (*n* = 5) for bacteriological and physicochemical analyses, and six fillets were allocated for consumer evaluation. All fillets were placed onto polystyrene trays and over-wrapped with oxygen-permeable 0.6 mm thick polyvinylchloride film (Delta Pack Ltd., São Paulo, Brazil). The trays were stored under refrigeration (4 ± 1 °C) and aerobic conditions in order to simulate retail conditions. On the last day of purchase, which corresponded to time zero of the storage, sensory, bacteriological, and physicochemical evaluations were performed on fish samples from each time point (days 8, 6, 4, 2 and 0). The sensory evaluation was performed by 104 consumers. All bacteriological and physicochemical analyses were performed in triplicate.

### 2.2. Consumers’ Sensory Evaluation

#### 2.2.1. Participants

A total of 104 consumers participated in the study (Table 1).

Consumers were recruited by convenience sampling, which does not represent a real population, but provides valuable qualitative inferences. Students, employees, and visitors from a research institution (Rio de Janeiro, Brazil) participated in the study, invited by telephone or e-mail, and the only criterion for selection was their willingness to participate in the study.

#### 2.2.2. Experimental Procedures

The fish fillets (from days 8, 6, 4, 2 and 0 of storage) were presented to participants in white plastic dish coded with three-digit numbers and in a balanced order of presentation. The consumers were instructed to visually rate how much they liked the color and the overall liking of the fish samples through a 9-point hedonic scale ranging from 1 (extremely disliked) to 9 (extremely liked) [28]. In addition, they answered whether they would accept or reject each sample by selecting “yes” or “no” for the questions (a) and (b) below [27]:

(a) “Supposing that this product is available on the market. Would you buy it?”

(b) “Supposing you bought this product and it is in your fridge. Would you consume it?”

Finally, they were asked to answer some socioeconomic questions (gender, age, education, and income) and their frequency of fish consumption. Data were collected using Compusense-Cloud (Compusense Inc., Guelph, ON, Canada). The test was performed on a single day, at the Brazilian Agricultural Research Corporation (Guaratiba, Rio de Janeiro/RJ). The sensory booths were designed in accordance with ISO 8589 [29], under artificial daylight and controlled temperature (22 °C).

### 2.3. Freshness Parameters

#### 2.3.1. Bacteriological Analyses

Serial dilutions of fish samples homogenates were inoculated through the pour-plate technique on the Plate Count Agar (PCA, Merck, Darmstadt, Germany) in Petri dishes to determine the total aerobic mesophilic count (TAMC) and the total aerobic psychrotrophic count (TAPC). The TAMC and TAPC were enumerated after incubation at 37 °C for 48 h and 10 °C for 7 days, respectively [30]. The results were expressed as log of CFU (colony-forming units)/g fish sample.

#### 2.3.2. Physicochemical Analyses

##### Lipid and Protein Oxidation

Lipid oxidation was evaluated using the 2-thiobarbituric acid reactive substances (TBARS) technique [31], adapted by Joseph et al. [32]. The absorbance values were recorded at 532 nm in a UV-1800 spectrophotometer (Shimadzu Corporation, Kyoto, Japan), and the results were expressed as mg malondialdehyde (MDA)/kg fish sample from a standard curve (R^2^ = 0.999) constructed with eight different MDA concentrations (0.5 to 400 µmol). Protein oxidation was evaluated by the methodology described by Oliver et al. [33], with modifications [34,35]. The protein carbonyl groups were detected and measured by reaction with 2,4 dinitrophenylhydrazine (DNPH). The absorbance of the samples was measured at 370 nm for carbonyl content and 280 nm for protein content using a UV-1800 spectrophotometer (Shimadzu Corporation, Kyoto, Japan). Results were expressed as nmol carbonyls/mg protein, based on the molar extinction coefficient of 21,000 M^−1^ cm^−1^.

##### Torrymeter Readings

The torrymeter readings were performed using the GR Torry Fish Freshness Meter (Distell Industries, West Lothian, Scotland). This equipment measures the dielectric properties of the tissues to assess the freshness of the samples. The sensors were applied directly onto three random locations on the surface of the fish fillets with temperatures between 0 and 10 °C [36]. Results were displayed on a convenient scale in the range of 0 (very spoiled) to 16 (very fresh) and expressed as the average values of the readings.

##### Instrumental Color Parameters’ Measurement

The instrumental color measurements were performed at three random locations on the surface of the fish fillets using a portable spectrophotometer CM-600D (Konica Minolta Sensing Inc., Osaka, Japan) with a measuring aperture diameter of 8 mm, illuminant A and 10° standard observer. The results were expressed as the average values of lightness (L*), redness (a*) and yellowness (b*). Additionally, the chroma (C*) and hue angle (H°) were calculated using the following equations [37]:

C* = [(a*)2 + (b*)2]1/2

H° = arctan (b*/a*)

##### Instrumental Texture Profile

Texture profile analysis (TPA) was performed in standardized size fillets (2 × 2 × 2 cm^3^) using a Texture Analyzer TA-XT plus (Stable Micro Systems, Surrey, UK) equipped with a 36 mm cylindrical probe (P/36). The TPA was conducted using two 50% compression cycles with 5 s intervals between compressions. The pre-test, test and post-test speeds were 1, 1 and 5 mm/s, respectively [38]. Hardness (HAD), chewiness (CWS), cohesiveness (CHS) and springiness (SPS) were calculated using the Exponent software package, version 6.1.9.1 (Stable Micro Systems, Surrey, UK).

### 2.4. Statistical Analyses

Survival analysis methodology (XLSTAT 2014.6.01, Addinsoft, Paris, France) was applied on consumer acceptance/rejection responses (questions “a” and “b”; no = 0; yes = 1) regarding two stages of the decision-making process (purchase and consumption) to estimate the sensory shelf life of Nile tilapia (*Oreochromis niloticus*) fillets stored at 4 ± 1 °C. The best data fit was obtained by applying a parametric survival regression model based on the Weibull distribution, using a 50% rejection level as a reference. The censoring of consumer data was performed in accordance with Hough et al. [27]. Results of consumers who rejected the fresh samples (day 0) were excluded from the study, resulting in a final data set of 92 consumers. The effect of storage time on the bacteriological and physicochemical parameters, as well as on the consumer acceptance, was assessed by one-way ANOVA and Duncan’s multiple range test (XLSTAT 2014.6.01, Addinsoft, Paris, France).

Consumer segmentation was based on purchase acceptance/rejection data over storage through means of hierarchical cluster analysis, following Euclidean distances and Ward’s method (Statistica^®^ 10, Statsoft, Tulsa, USA). Then, survival analysis methodology was applied on cluster data, as previously described. Frequency counts were tabulated for consumers’ socioeconomic characteristics, and the chi-squared test per cell was conducted to assess the significant associations and trends between the consumer clusters [39]. A partial least square regression (PLSR) was performed to identify which freshness parameters contributed positively or negatively to consumers’ purchase rejection before and after consumer segmentation. A confidence level of 95% was considered for all analyses.

## 3. Results and Discussion

### 3.1. Consumers’ Evaluation

#### 3.1.1. Consumers’ Acceptability Limit Based on Survival Analysis

**Figure 1:**
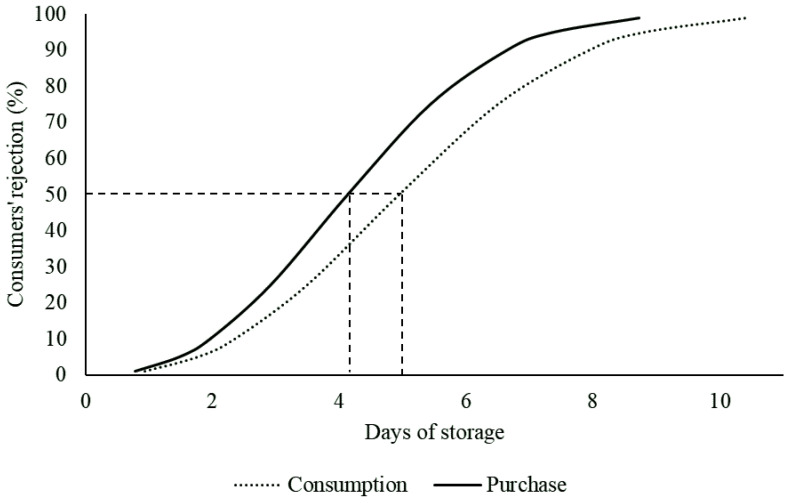
Probability of consumers’ rejection for the consumption and purchase of Nile tilapia (*Oreochromis niloticus*) fillets versus storage time at 4 ± 1 °C by Weibull distribution; 50% rejection points are indicated by the dashed line.

The Weibull distribution revealed that the acceptability limit of refrigerated Nile tilapia (*Oreochromis niloticus*) fillets was estimated to be 4.128 (μ = 1.563 and σ = 0.395) and 4.946 (μ = 1.743 and σ = 0.394) days for purchase and consumption, respectively, considering a 50% consumer rejection probability (Figure 1).

These results indicate, as expected, that consumers are more rigorous when selecting a product at purchase than at the consumption stage. According to Giménez et al. [40], consumers are more tolerant when they have already bought the product because they do not want to discard it. This behavior can be triggered by economic, social and/or environmental concerns [41]. Therefore, considering the aims of the present study, only the acceptability limit at the time of purchase was used as a reference for carrying out the following discussion.

#### 3.1.2. Survival Analysis Versus Hedonic Scores for Assessing Consumers’ Acceptability Limits

Consumers are usually unable to touch or smell fish fillets at retail; therefore, fish freshness evaluations are commonly based on visual cues such as color, shape, and surface characteristics. Consumers’ hedonic reactions towards appearance and specific sensory traits of the products can be measured by asking their degree of liking using a hedonic scale [42]. Regarding fish and fish products, the scores assigned by consumers usually decrease over storage due to the occurrence of deteriorative processes that negatively affect their appearance [43,44]. Accordingly, in the present study, the overall liking (OL) and color liking (CL) scores of Nile tilapia (*Oreochromis niloticus*) decreased (*p* < 0.05) when comparing day 0 (OL: 7.99 ± 1.04; CL: 7.80 ± 1.38) with day 8 (OL: 2.98 ± 2.18; CL: 3.02 ± 2.14) of refrigerated storage. Nonetheless, overall liking scores revealed information regarding consumer dissatisfaction, and not necessarily the consumer rejection of the product [16].

In this way, the determination of acceptability limits based on hedonic scores requires the selection of a cut-off point, which may differ among researchers [45]. Monteiro et al. [24] and Rong et al. [43] selected a score of 4, whereas Moawad et al. [46] selected a score of 5 as the acceptability limit for refrigerated Nile tilapia (*Oreochromis niloticus*) fillets in a 9-point hedonic scale. Nonetheless, a score of 5 is more suitable to be used as a cut-off point, once point 4 is in the dislike range in the aforementioned scale. In the present study, the OL and CL scores on day 4 of storage were 5.74 ± 2.17 and 5.48 ± 2.24, whereas on day 6, they were 4.00 ± 2.20 and 3.70 ± 1.98, respectively. Therefore, applying the previously suggested cut-off point, the consumers’ acceptability limit would agree with that obtained by the survival analysis methodology.

### 3.2. Freshness Parameters and Their Relationship with Consumers’ Purchase Rejection

Bacteriological, physicochemical, and instrumental color and texture parameters were compared, considering the last day of storage in which the samples were accepted (day 4; D4) and the first day of storage in which the samples were rejected (day 6; D6) by consumers, according to survival analysis.

#### 3.2.1. Bacteriological Parameters

Bacterial growth directly impacts the safety of fish consumption, in addition to being considered the most important cause of fish spoilage and quality loss, which is commonly observed during refrigerated storage [38,47]. Likewise, the total aerobic mesophilic count (TAMC) and total aerobic psychrotrophic count (TAPC) increased (*p* < 0.05) over storage, including from D4 to D6 (Table 2).

According to the International Commission on Microbiological Specifications for Foods [48], fish is considered unfit for consumption when the TAPC reaches 7 log CFU/g. In our study, refrigerated Nile tilapia (*Oreochromis niloticus*) fillets exceeded this limit on day 4, when consumers still considered them suitable for purchase by the survival analysis results. These results are alarming because the metabolites resulting from microbial growth, in addition to causing sensory alterations, are toxic and may cause foodborne illnesses [49,50]. Moreover, they also highlight the importance of developing sensors that indicate quality through the detection of compounds arising from degradation [22].

#### 3.2.2. Lipid and Protein Oxidation

Inherent fish characteristics, such as the high amount of unsaturated fatty acids and proteins, increase the susceptibility to oxidation damage [51]. Lipid and protein oxidation occur in a similar pathway based on complex free radical chain reaction, which starts immediately after slaughtering, mainly due to the attenuation of the endogenous antioxidant defense mechanisms and to the increased exposure to oxidation agents such as oxygen, light, temperature, metals, and enzymes [52]. Both lipid and protein oxidation lead to the development of secondary products that cause unpleasant alterations in sensory attributes such as color, flavor, and texture, representing some of the main limitations for consumers’ acceptability of fish [53].

Although only carbonyl levels increased (*p* < 0.05) from D4 to D6, both carbonyl and malondialdehyde (MDA) levels increased (*p* < 0.05) over storage (Table 2). The connection between the mechanisms of lipid and protein oxidation is well-recognized, which indicates that they start together and can interact with each other [54]. Monteiro et al. [38] also observed this pattern during the refrigerated storage of tilapia fillets. Dergal et al. [55] proposed a threshold of 0.85 mg MDA/kg for Nile tilapia, corresponding to the value at which fish were rejected by the sensory control. In the present study, refrigerated Nile tilapia (*Oreochromis niloticus*) fillets did not exceed this limit during the entire storage period. Despite the relevance of protein oxidation for food quality, there are no suggested limits based on sensory rejection for carbonyl levels in fish.

#### 3.2.3. Torrymeter Readings

A torrymeter is used to instrumentally evaluate the freshness of fish by measuring the modified dielectric properties of the tissue [56]. The dielectric properties of fish skin and muscle are altered due to the cellular structure degradation caused by bacteriological and enzyme activity during spoilage. Therefore, modifications on torrymeter readings are mainly associated with alterations in texture and have been used as quality indicators [36].

In the present study, the torrymeter readings decreased (*p* < 0.05) from D4 to D6 (Table 2). Similar trends were observed by Ochrem et al. [57] in chilled carp (*Cyprinus carpio*), and by Sant’Ana et al. [58] in ice-stored blackspot seabream (*Pagellus bogaraveo*). According to the equipment manufacturer, scores below 4 indicate that fish is not suitable for consumption. Nonetheless, in the present study, this score was never reached, even when the Nile tilapia (*Oreochromis niloticus*) fillets were rejected by consumers (D6) or reached the suggested bacterial limit (D4). Therefore, despite indicating the decline in freshness over storage time, the torrymeter readings were not sufficient to predict quality limits, which was also observed by Badiani et al. [59] in cuttlefish (*Sepia officinalis* L.) under iced storage.

#### 3.2.4. Instrumental Color Parameters

Among the specific characteristics that contribute to the appearance of fish and other meat types, color is the one that most influences consumers’ choice [60]. L*, b*, C* and H° values increased, whereas a* values decreased during storage (*p* < 0.05). Moreover, only a* values were different (*p* < 0.05) when comparing D4 and D6 (Table 2). In agreement with our results, Chen et al. [61] evaluated the quality of Nile tilapia (*Oreochromis niloticus*) fillets during ice storage and reported a decrease in a* values. They also observed that redness was the only color parameter that presented a significant difference between the last day of sensory acceptance and the first day of unacceptance; however, it was based on a 5-point rating scale (5 = desirable; 1 = extremely unacceptable). A decrease in redness during storage is well known and suggests browning of the fish surface due to the oxidation of myoglobin into metmyoglobin [37].

The increases in lightness and yellowness were previously reported in freshwater fish species stored under refrigeration [62,63]. These results may be associated with changes in the light scattering due to protein denaturation, and consequent increases in unbound water [64], and with the increase in yellow pigments yielded from the oxidation of lipids and proteins [65]. Regarding C* and H° values, a similar trend was observed by Joseph et al. [62] in sunshine bass during refrigerated storage. C* and H° were calculated and interpreted from a* and b* values. Thus, a progressive increase in C* during storage indicated that the increase in b* values was numerically more pronounced than the decrease in a* values. Moreover, greater H° values means greater deviation from red color over the storage period [37].

#### 3.2.5. Texture Profile Analysis

Fish texture is considered to be important quality attribute for palatability and exerts a considerable effect on consumers’ purchase decisions [66]. Hardness and chewiness decreased (*p* < 0.05) when comparing D4 and D6, whereas cohesiveness and springiness remained stable over storage (*p* > 0.05) (Table 2). Monteiro et al. [38] and Saéz et al. [67] also observed a decrease in hardness and chewiness in tilapia and rainbow trout fillets under refrigerated storage, respectively. These results indicate that the fish fillets became softer over storage due to the activity of endogenous and microbial proteolytic enzymes, which induce the degradation of myofibrillar proteins and the disruption of the connective tissue [68]. Regarding cohesiveness and springiness, they describe the resistance and the ability of muscles to recover their original form after deformation. In agreement with our results, Saéz et al. [67] reported that storage time did not affect these parameters in rainbow trout fillets.

### 3.3. Multivariate Analysis of Freshness Parameters and Consumers’ Rejection

**Figure 2:**
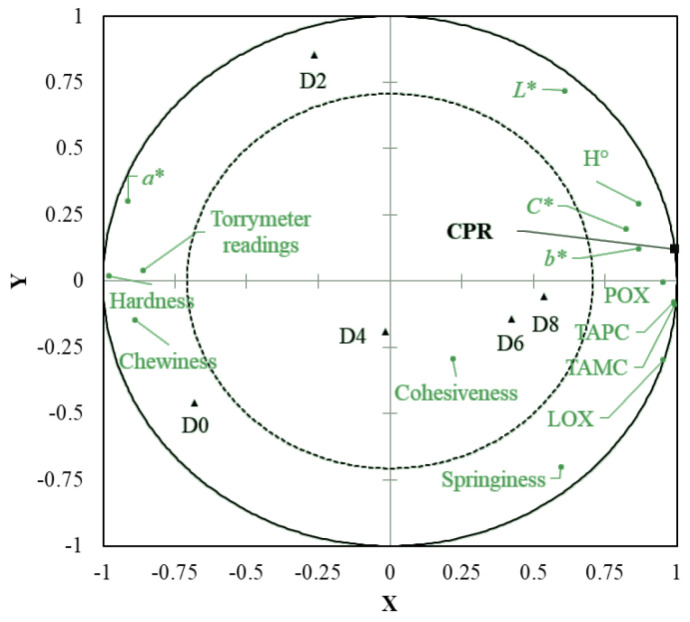
Partial least square regression (PLSR) model for the freshness parameters of Nile tilapia (*Oreochromis niloticus*) fillets stored at 4 ± 1 °C for 8 days. X axis = freshness parameters; Y axis = consumer’s purchase rejection (CPR). LOX—lipid oxidation; POX—protein oxidation; C*—chroma; H°—hue angle; TAMC—Total aerobic mesophilic count; TAPC—total aerobic psychrotrophic count.

The partial least square regression (PLSR) model (Figure 2) explained 100% of consumer purchase rejection (*Y* axis) and 82.1% of the freshness parameters (*X* axis), yielding an accumulated Q^2^ of 0.997.

The freshness parameters were considered relevant when their respective variable importance projection (VIP) values were greater than 1.0 [69]. Among the parameters evaluated, only L*, C*, cohesiveness and springiness were not retained as determinants for consumers’ rejection decisions. The TAMC, TAPC, protein oxidation, lipid oxidation, H° and b* parameters were positively correlated with consumer purchase rejection, whereas hardness, chewiness, a* and torrymeter readings presented a negative correlation.

Among these parameters, TAMC, TAPC, protein oxidation, torrymeter readings, a*, hardness and chewiness values differed from D4 to D6, indicating that they were the main freshness indicators influencing fish rejection. Furthermore, the changes in a* values presented the greatest contribution to consumer rejection when compared with other color parameters.

On the other hand, although lipid oxidation, H° and b* values influenced the consumers’ purchase rejection, they did not (*p* > 0.05) present individual differences between accepted (D4) and rejected (D6) samples. These results reinforce the idea that consumers’ decisions are mostly based on multidimensional criteria, rather than on specific product traits. In addition to intrinsic food factors, individual consumer characteristics strongly influence the decision to accept or reject a product [15].

### 3.4. Consumers’ Acceptability Limits Are Driven by Socioeconomic Characteristics and Fish Consumption Frequency

Two groups of consumers clearly differed in purchase rejection patterns and socioeconomic characteristics in terms of gender, education, and income (*p* < 0.05; Table 3).

**Figure 3:**
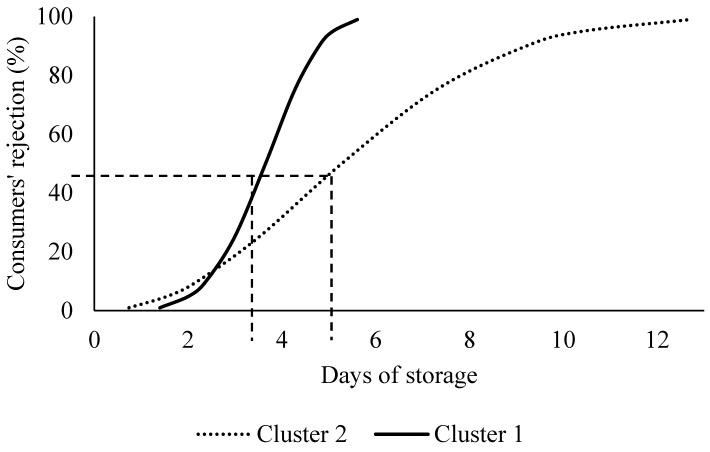
Probability of clusters 1 and 2 consumers’ rejection for the purchase of Nile tilapia (Oreochromis niloticus) fillets versus storage time at 4 ± 1 °C by Weibull distribution; 50% rejection points are indicated by the dashed line.

There were no differences (*p* > 0.05) regarding age and fish consumption frequency. Cluster 1 (*n* = 62; 67,4% of total consumers) presented a higher number of women (*p* >0.05) and graduate consumers, whereas cluster 2 (*n* = 30; 32,6% of total consumers) presented significantly more men and consumers from lower educational levels. Income differed only in some levels between the two clusters, with a tendency for lower-income consumers to belong to cluster 2. Cluster 1 was characterized by an early rejection, presenting 48.33%, 63.33%, 93.33% and 100% of consumers rejecting the samples on days 2, 4, 6 and 8 of storage, respectively. Cluster 2 was characterized by a late rejection, where the percentage of consumers rejecting the samples on days 2, 4, 6 and 8 were 20%, 16.67%, 56.67% and 63.33%, respectively. In relation to survival analysis, it revealed that the acceptability limit for purchase of refrigerated Nile tilapia (*Oreochromis niloticus*) fillets for consumers of cluster 1 was estimated at 3.650 (μ = 1.378 and σ = 0.227), and for cluster 2, it was 5.275 (μ = 1.833 and σ = 0.464) days (Figure 3).

In general, women and people with higher education levels are more aware of the importance of fish quality assessments for general health. These segments usually seek reliable information from professionals to make safer fish choices [70]. Moreover, men tend to be less concerned than women with the possible negative effects of food and place less importance on freshness attributes [71].

Income and education are highly correlated; therefore, variations in these segments tend to demonstrate similar behaviors. Low-income and less educated consumers usually present low motivation towards healthy eating and attach less importance to quality [72]. Therefore, their purchasing decisions tend to be based on other attributes, rather than on fish appearance [12]. Obviously, the high price of fish is perceived as a barrier for these consumers, who can accept low-quality products because they are cheaper choices [73].

**Figure 4:**
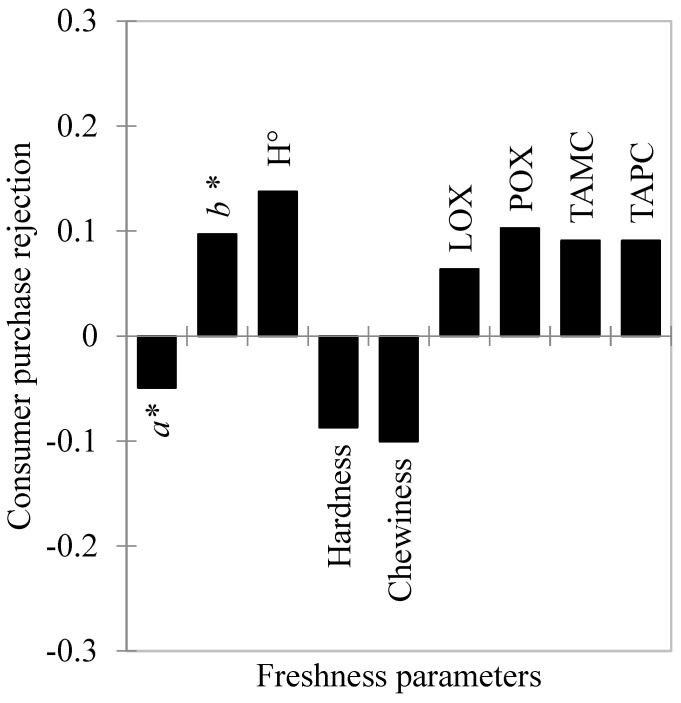
Weighted regression coefficients of cluster 1 for freshness parameters of Nile tilapia (*Oreochromis niloticus*) fillets stored at 4 ± 1 °C for 8 days by partial least square regression (PLSR). X axis = freshness parameters; Y axis = consumer purchase rejection (CPR). a*—redness; b*—yellowness; H°—hue angle; LOX—lipid oxidation; POX—protein oxidation; TAMC—total aerobic mesophilic count; TAPC—total aerobic psychrotrophic count.

**Figure 5:**
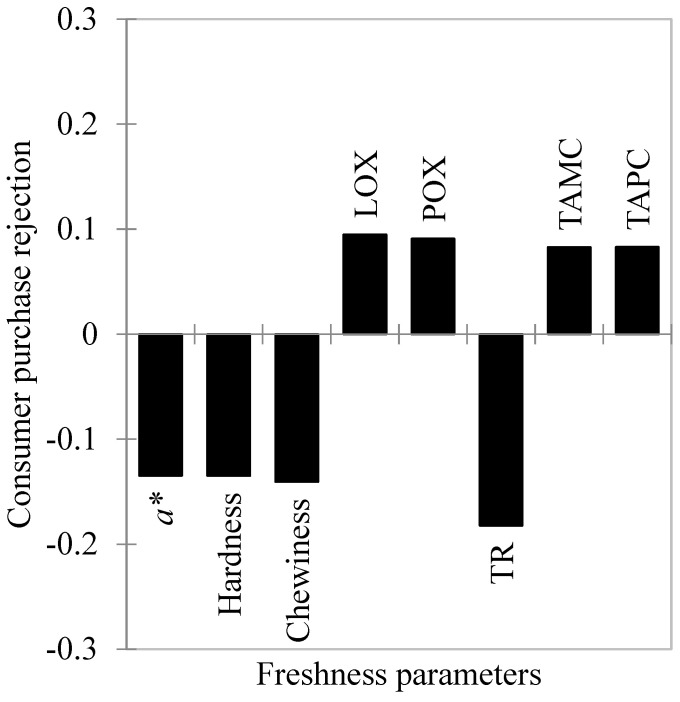
Weighted regression coefficients of cluster 2 for freshness parameters of Nile tilapia (*Oreochromis niloticus*) fillets stored at 4 ± 1 °C for 8 days by partial least square regression (PLSR). X axis = freshness parameters; Y axis = consumer purchase rejection (CPR). a*—redness; LOX—lipid oxidation; POX—protein oxidation; TR—torrymeter readings; TAMC—total aerobic mesophilic count; TAPC—total aerobic psychrotrophic count.

Partial least square regression (PLSR) was also conducted separately for both clusters (Figure 4 and Figure 5).

For cluster 1, the PLSR model explained 100% of consumer purchase rejection (*Y* axis) and 82% of the freshness parameters (*X* axis), yielding an accumulated Q^2^ of 0.974. For cluster 2, the PLSR model explained 100% of consumer purchase rejection (*Y* axis) and 83% of the freshness parameters (*X* axis), yielding an accumulated Q^2^ of 0.990. The parameters that differed between the two segmented groups were b* and H° that were considered important for cluster 1, but not for cluster 2; torrymeter readings were important for cluster 2, but not for cluster 1. Moreover, most parameters did not contribute to the same degree for both clusters. Based on the regression analyses, three parameters (a*, b* and H°) of high importance for cluster 1 were directly associated with color, whereas there was only one (a*) for cluster 2. Furthermore, the three most important parameters for cluster 2 were directly related to texture (torrymeter readings, chewiness, and hardness). It was assumed, therefore, that consumers in cluster 1 mainly based their choices on the discoloration of Nile tilapia fillets, whereas for consumers in cluster 2, the dominant aspect in the perception of freshness was the consistency of the fillets. In addition, differently from cluster 1, the least important parameters for cluster 2 were TAMC and TAPC, suggesting a greater difficulty for this group to visually estimate the bacteriological safety of the fillets.

These results indicate that consumers with profiles similar to cluster 2 are more likely to buy spoiled fillets because the bacteriological limit indicated for safe consumption was reached on day 4 of storage. This behavior can lead to greater post-purchase dissatisfaction because, in addition to the increased risk of foodborne diseases, there is also the accumulation of compounds responsible for off-flavors in fish [8,12]. On the other hand, the early rejection of fresh fish by consumers such as those in cluster 1 is worrying due to increases in economic loss and food waste at retail [7], which have been identified by the FAO [1] as one of the main problems to fish market expansion.

## 4. Conclusions

The present study demonstrated that the multivariate approach was more suitable to evaluating fish spoilage and consumers’ purchase rejection than using multiple freshness indicators singly. It was also observed that socioeconomic characteristics of consumers, such as gender, education, and income, affected the assessment and interpretation of the intrinsic attributes related to the fish freshness. Nonetheless, the influence of different frequencies of fish consumption and age of consumers on the assessment of fish freshness was not clarified. This result may be related to the limitations imposed by our sample of consumers, and also by the scale used to verify these parameters in the present study. Therefore, ignoring the heterogeneity of consumers can lead to the loss of important data. This information should be considered when developing new studies and educational campaigns related to fish consumption, which are most effective when adapted to the needs of well-defined target audiences.

In addition, investments must be made in the development of technologies that facilitate the assessment of fish quality at the point of sale and that are accessible to all types of consumers. The responsibility for ensuring safe and healthy products at the point of sale must lie with producers and distributors. However, improving consumers’ ability to make good choices when buying fresh fish would bring social and economic benefits related to public health and to the seafood industry, which would enable them to make relevant claims and demand their rights.

## Figures and Tables

**Table 1:** Socioeconomic information of the consumers (n = 104).

	Consumers
N	104
*Gender (%)*	
Female	54.8
Male	45.2
*Age (years) (%)*	
18–25	9.6
26–35	31.7
36–45	36.5
46–55	10.6
56–65	9.6
>65	1.9
*Education (%)*	
Primary school	1.9
Secondary school	18.3
Under graduation	16.3
Graduation	63.5
*Income—minimum Brazilian wage (BRL 998.0)*^a^ (%)	
1 to 5	26.0
>5 to 10	27.9
>10 to 20	28.8
>20 to 30	11.5
>30	5.8

^a^ In Brazilian currency (BRL).

**Table 2:** Freshness parameters of Nile tilapia (*Oreochromis niloticus*) fillets stored at 4 ± 1 °C for 8 days.

Freshness Parameters	Days of Storage				
	0	2	4	6	8
*Bacteriological* ^€^					
Total aerobic mesophilic count (TAMC)	4.94 ± 0.47 ^e^	5.82 ± 0.32 ^d^	**6.92 ± 0.24** ^c^	**7.93 ± 0.23** ^b^	8.60 ± 0.21 ^a^
Total aerobic psychrotrophic count (TAPC)	5.5 ± 0.17 ^e^	6.41 ± 0.44 ^d^	**7.47 ± 0.53** ^c^	**8.43 ± 0.24** ^b^	9.24 ± 0.46 ^a^
*Physicochemical*					
Lipid oxidation *	0.03 ± 0.00 ^c^	0.03 ± 0.01 ^c^	**0.04 ± 0.01** ^b^	**0.05 ± 0.01** ^ab^	0.05 ± 0.01 ^a^
Protein oxidation ^#^	5.52 ± 0.92 ^d^	6.76 ± 1.28 ^c^	**7.57 ± 1.25** ^bc^	**9.15 ± 1.84** ^a^	8.27 ± 1.03 ^ab^
Torrymeter readings	11.14 ± 0.92 ^a^	10.64 ± 0.93 ^a^	**11.33 ± 1.05** ^a^	**8.34 ± 1.64** ^b^	7.73 ± 1.11 ^b^
*Color*					
Lightness (L*)	47.35 ± 2.31 ^b^	51.69 ± 1.30 ^a^	**50.72 ± 1.15** ^a^	**50.57 ± 1.64** ^a^	50.82 ± 1.28 ^a^
Redness (a*)	2.10 ± 0.38 ^ab^	2.19 ± 0.39 ^a^	**1.81 ± 0.37** ^b^	**0.89 ± 0.10** ^c^	1.00 ± 0.32 ^c^
Yellowness (b*)	1.64 ± 0.33 ^c^	2.65 ± 0.71 ^b^	**3.02 ± 0.69** ^b^	**2.90 ± 0.90** ^b^	4.51 ± 1.37 ^a^
Chroma (C*)	5.08 ± 0.87 ^c^	8.31 ± 1.66 ^b^	**8.29 ± 0.47** ^b^	**8.30 ± 0.93** ^b^	13.56 ± 2.62 ^a^
Hue angle (H°)	0.57 ± 0.09 ^c^	1.07 ± 0.20 ^b^	**1.18 ± 0.19** ^ab^	**1.31 ± 0.15** ^a^	1.18 ± 0.14 ^ab^
*Texture*					
Hardness (N)	10.33 ± 2.48 ^a^	8.97 ± 2.71 ^a^	**8.60 ± 2.79** ^a^	**5.80 ± 1.38** ^b^	5.88 ± 1.27 ^b^
Chewiness (N × mm)	5.45 ± 1.58 ^a^	4.50 ± 1.44 ^abc^	**4.66 ± 1.16** ^ab^	**3.19 ± 0.72** ^c^	3.95 ± 0.92 ^bc^
Cohesiveness (ratio)	0.69 ± 0.02 ^a^	0.69 ± 0.02 ^a^	**0.71 ± 0.03** ^a^	**0.70 ± 0.04** ^a^	0.69 ± 0.03 ^a^
Springiness (ratio)	0.89 ± 0.08 ^a^	0.87 ± 0.07 ^a^	**0.91 ± 0.05** ^a^	**0.90 ± 0.06** ^a^	0.91 ± 0.04 ^a^

Results are expressed as the mean ± standard deviation (*n* = 5). Means without common superscripts (a, b, c, d and e) in a row are different (*p* < 0.05). ^€^, Expressed as log CFU (colony-forming units)/g. *, Expressed as milligrams of malondialdehyde per kilogram of sample. ^#^, Expressed as nanomoles of carbonyl per milligram of protein. Day 4 in bold: last storage time where the samples were accepted by consumers, considering the 50% limit of rejection. Day 6 in bold: first storage time where the samples were rejected by consumers, considering the 50% limit of rejection.

**Table 3:** Socioeconomic characteristics of consumers from clusters 1 and 2 (*n* = 92).

Socioeconomic Characteristics	Consumers	
	Cluster 1	Cluster 2
N	62	30
*Gender (%)*		
Female	65 (+) **	33 (−) **
Male	35 (−) **	67 (+) **
*Education (%)*		
Primary school	0 (−) *	7 (+) *
Secondary school	0 (−) ***	53 (+) ***
Undergraduate	10 (−) *	30 (+) *
Graduation	90 (+) ***	10 (−) ***
*Income—minimum Brazilian wage (BRL 998.00)*^a^ (%)		
1 to 5	16 (−) **	47 (+) **
>5 to 10	27	30
>10 to 20	35 (+) *	13 (−) *
>20 to 30	15	7
>30	6	3

^a^ In Brazilian currency (BRL). Effect of the chi-square per cell. (+) or (−) indicate that the observed value is higher or lower than the expected theoretical value, respectively: * *p* < 0.05; ** *p* < 0.01; *** *p* < 0.001.

## Data Availability

The data of the present study are available from the corresponding author upon request.
